# Morbidity and mortality reduction in acute decompensated heart failure: a result of individualized monitoring or intervention principles?

**DOI:** 10.1093/eschf/xvag217

**Published:** 2026-08-12

**Authors:** Ahmed B Shamsulddin

**Affiliations:** Algibejee Primary Health Care Center, Baghdad, Iraq

**Keywords:** Acute decompensated heart failure, Diuresis, Natruresis, Urine output

To the Editor

We read with great interest the pragmatic, real-world evaluation by Geraeds et al.^1^ which demonstrates that a natriuresis- and urine-output-guided protocol for acute decompensated heart failure (ADHF) significantly reduces length of hospital stay, cardiac rehospitalizations, and mortality. The authors confirm that tailoring loop diuretics based on early spot urine sodium levels drives these impressive clinical outcomes. While we are impressed by the successful implementation of a standardized decongestion algorithm, a closer examination of the protocol’s mechanics suggests that the true driver of success may lie not in how we monitor these patients, but in how we deliver the drug.

The authors’ guided protocol mandates an initial loop diuretic bolus followed directly by a continuous intravenous (cIV) infusion of bumetanide. Under standard care, patients typically receive intermittent intravenous (iIV) boluses. As demonstrated by Thomson et al., continuous infusions provide significantly more efficient diuresis and greater total urine output compared to intermittent dosing.^2^ Therefore, the shortened length of stay observed by Geraeds et al. may be primarily attributable to the pharmacokinetic superiority of continuous infusion, rather than the natriuresis-guided dose titrations alone.

Furthermore, the utilization of continuous infusions introduces a fascinating, often-overlooked physiological variable: the obligate administration of the IV carrier fluid. While it seems inherently paradoxical to administer fluids to a hypervolemic patient, recent evidence suggests otherwise. As Iwanek et al. surprisingly demonstrated, IV sodium chloride infusion during ADHF decongestion is actually associated with increased net natriuresis and augmented diuresis.^3^ The steady, low-dose administration of sodium chloride and volume accompanying a continuous diuretic infusion likely preserves intravascular volume and renal perfusion, which in turn leads to higher urine output.

We commend Geraeds et al. for proving that standardized, intensive diuretic protocols save days and lives. However, we must ask whether the hero of this study is the spot urine sodium check, or simply the physiological elegance of continuous, fluid-supported loop diuretic delivery. The answer to this question is not simple, instead it is crucial, as it will ultimately shape future guidelines—determining whether the standard of care should mandate continuous infusion regimens, natriuresis-guided titration, or a synthesis of both.

## Declarations

### Data Availability

No data were generated or analysed for this manuscript.
